# Reporting remit and function of trial steering committees in randomised controlled trials: review of published literature

**DOI:** 10.1186/1745-6215-16-S2-P177

**Published:** 2015-11-16

**Authors:** EJ Conroy, B Arch, S Lewis, A Lane, MR Sydes, J Norrie, G Murray, NL Harman, C Gamble

**Affiliations:** 1 Medicines for Children Clinical Trials Unit, University of Liverpool, Liverpool, UK; 2 Centre for Population Health Sciences, University of Edinburgh, Edinburgh, UK; 3 Bristol Randomised Trials Collaboration Trials Unit, Bristol, UK; 4 MRC Clinical Trials Unit at UCL, London, UK; 5 London Hub for Trials Methodology Research, London, UK; 6 Centre for Healthcare Randomised Trials (CHaRT), Aberdeen, UK; 7 MRC North West Hub for Trials Methodology Research, Department of Biostatistics, University of Liverpool, Liverpool, UK

## Background

DAMOCLES established a Data Monitoring Committee (DMC) Charter which has been widely used for RCTs. The DMC is typically advisory and makes recommendations to another executive body; the Trial Steering Committee (TSC). Despite the executive role of the TSC over the DMC, the CONSORT statement doesn't explicitly require reporting of TSC activity although TSCs are included as an example of good reporting.

## Methods

A cohort of published RCTs was examined to identify reporting practice. The cohort included three key journals and the HTA monograph series. Main publications, supplementary documentation, including protocols, were searched. Reporting of TSC membership, organisation and role were extracted with impact on trial design, conduct and analysis.

## Results

144/264 publications reported a TSC. Reporting TSC existence and input varied between journals with many of the publications only reporting an acknowledgment. Terminology used to describe a TSC was inconsistent this meant that distinguishing the executive body from the DMC and the day-to-day Trial Management Group was difficult. Protocols published as a supplementary document improved reporting.

## Conclusion

TSC existence and activity is underreported. Quality of reporting and variation in terminology does not facilitate transparency of trial oversight structure. Descriptions of committee structures and remits are absent. Supplementary materials could be used to improve reporting including mandatory publication of protocols. CONSORT should make recommendations to improve reporting of such committees extending their remit to include supplementary materials as well as the main article.

